# Meta-Analysis: Melatonin for the Treatment of Primary Sleep Disorders

**DOI:** 10.1371/journal.pone.0063773

**Published:** 2013-05-17

**Authors:** Eduardo Ferracioli-Oda, Ahmad Qawasmi, Michael H. Bloch

**Affiliations:** 1 University of São Paulo Medical School, São Paulo, Brazil; 2 Yale Child Study Center, Yale University, New Haven, Connecticut, United States of America; 3 Children’s Hospital of Michigan, Detroit, Michigan, United States of America; 4 Department of Psychiatry of Yale University, New Haven, Connecticut, United States of America; St. Joseph’s Hospital and Medical Center, United States of America

## Abstract

**Study Objectives:**

To investigate the efficacy of melatonin compared to placebo in improving sleep parameters in patients with primary sleep disorders.

**Design:**

PubMed was searched for randomized, placebo-controlled trials examining the effects of melatonin for the treatment of primary sleep disorders. Primary outcomes examined were improvement in sleep latency, sleep quality and total sleep time. Meta-regression was performed to examine the influence of dose and duration of melatonin on reported efficacy.

**Participants:**

Adults and children diagnosed with primary sleep disorders.

**Interventions:**

Melatonin compared to placebo.

**Results:**

Nineteen studies involving 1683 subjects were included in this meta-analysis. Melatonin demonstrated significant efficacy in reducing sleep latency (weighted mean difference (WMD) = 7.06 minutes [95% CI 4.37 to 9.75], Z = 5.15, p<0.001) and increasing total sleep time (WMD = 8.25 minutes [95% CI 1.74 to 14.75], Z = 2.48, p = 0.013). Trials with longer duration and using higher doses of melatonin demonstrated greater effects on decreasing sleep latency and increasing total sleep time. Overall sleep quality was significantly improved in subjects taking melatonin (standardized mean difference = 0.22 [95% CI: 0.12 to 0.32], Z = 4.52, p<0.001) compared to placebo. No significant effects of trial duration and melatonin dose were observed on sleep quality.

**Conclusion:**

This meta-analysis demonstrates that melatonin decreases sleep onset latency, increases total sleep time and improves overall sleep quality. The effects of melatonin on sleep are modest but do not appear to dissipate with continued melatonin use. Although the absolute benefit of melatonin compared to placebo is smaller than other pharmacological treatments for insomnia, melatonin may have a role in the treatment of insomnia given its relatively benign side-effect profile compared to these agents.

## Introduction

Primary sleep disorders are those not associated with a medical condition, substance use or concurrent psychological disorder. In order to be diagnosed with a primary sleep disorder, the sleep disturbance must cause significant distress or impairment in social, occupational, or other areas of functioning [1]. Nine percent of Americans report having insomnia [2]. Thirty-five to forty percent of Americans report having problems falling asleep or excessive daytime sleepiness [3]. Primary sleep disorders are often comorbid with psychiatric disorders, neurological and cardiovascular diseases [4]. Average medical expenses of individuals with insomnia in the United States is nearly $2000 greater annual than those without sleep problems [5]. Poor sleep is also associated with an increased risk of mortality, hospitalization and traffic accidents [6]–[8].

First line treatment options for primary sleep disorders often include psychological or behavioral therapies [9]. Sleep hygiene, sleep restriction, stimulus control, relaxation training and cognitive therapy are examples of such non-pharmacological therapies, all of which have shown some evidence of efficacy [10]–[12]. Pharmacological treatments for primary sleep disorders, like insomnia, include benzodiazepine receptor agonists, benzodiazepines, sedating antidepressants and other drugs with sedating feature (anxiolytics, antipsychotics, antihistamines). Side-effects vary between these medications and can range from residual daytime sleepiness to dependence [13]. There are many over-the-counter medications and herbal therapies that are used by individuals to treat insomnia. However the efficacy and side-effect profile of these substances are not as well known or studied as prescription medications [14].

Many trials have been performed to assess the efficacy of exogenous melatonin in treating primary sleep disorders. Melatonin is a hormone secreted primarily by the pineal gland in response to variations in the circadian cycle and has been used for the last two decades for the treatment of sleep disorders in adults and children [15]. In contrast to most available sleep medications, melatonin has little dependence potential, is not associated with habituation and typically produces no hangover. Given its reported hypnotic effects, relatively benign side-effect profile and over-the-counter availability, melatonin has been widely utilized in the United States [16].

A previous meta-analysis demonstrated that melatonin was beneficial in treating most primary sleep disorders over the short-term (4 weeks or less) [17]. However, since this meta-analysis, 7 trials have been published with an additional 1258 subjects. These additional trials nearly triple the sample size of previous meta-analyses. The additional power provided by these new trials will (1) allow us to more precisely estimate measures of treatment effects and (2) examine moderators of melatonin efficacy. Specifically, we will examine melatonin’s effects on sleep latency, total sleep time and sleep quality. We will also examine the moderating effects of measure type, dose and duration of melatonin treatment.

## Methods

### Selection of Studies

PubMed was searched by two reviewers (AQ and EFO) using the terms “Melatonin” and “Sleep Disorder”. The search was further limited to include only randomized controlled trials and meta-analyses. The bibliographies of related reviews, meta-analyses and included articles were searched for additional eligible citations. All studies included were published before or on March 2012.

### Inclusion Criteria

Trials were included if they (1) analyzed primary sleep disorders as defined by the DSM-IV, (2) examined the effects of melatonin, (3) were randomized placebo controlled trials, (4) had at least 10 participants for parallel designs or 5 participants for crossover designs and (5) were published in English. Disagreements regarding the inclusion of studies were discussed between the two reviewers (AQ and EFO) and ultimately decided by the third reviewer (MHB) when necessary.

### Meta-analytic Procedures

Data was extracted using Microsoft Excel spreadsheets. Extracted data included sleep onset latency, total sleep time, sleep quality, age of sample, dose, duration, drug formulation. Sleep onset latency, total sleep time and sleep quality data were also classified in objective measures (polysomnography or actigraphy) and/or subjective (scales, questionnaires, sleep logs).

Our primary outcome measure was mean improvement in sleep onset latency, total sleep time and sleep quality. In this meta-analysis, we considered sleep efficacy the same as sleep quality. We examined the difference between melatonin and placebo by calculating the weighted mean difference (WMD) using Comprehensive Meta-Analysis (Biostat, Englewood, NJ) for sleep latency and total sleep time analysis. Sleep quality was analyzed in Comprehensive Meta-Analysis by calculating the standardized mean difference (SMD). SMD was favored over WMD for measuring sleep quality because rating scales assessing sleep quality differed between the included studies. A fixed-effects model was used for this meta-analysis with the results for a random-effects model presented as a sensitivity analysis.

Publication bias was assessed by plotting the effect size against standard error for each trial (funnel plot) [18]. In addition, publication bias was statistically tested by the Egger’s test and by determining the association between sample size and effect size in meta-regression [18]. Heterogeneity between trials was determined by means of two separate statistical estimates using Comprehensive Meta-Analysis. First, a *Q-*statistic was employed to provide a test of statistical significance indicating whether the differences in effect sizes are due to subject-level sampling error alone or other sources. In addition, we estimated heterogeneity using *I-square* statistic, which estimates the proportion of total variance that is attributable to between-study variance.

For secondary analyses we performed several subgroup analyses and meta-regressions. Stratified subgroup analysis was used to assess the effects of type of measure (subjective/objective). We used the test for subgroup differences in Comprehensive Meta-Analysis to determine whether subgroups reduced overall heterogeneity [19]. We initially intended to examine the effects of age group (children younger than 18 years old vs. adults older than 18 years old) on melatonin’s effects. However, there were not enough trials in children to conduct this analysis. Meta-regression was performed to examine the association between melatonin efficacy in trials and continuous variables such as (1) dose and (2) duration. Our threshold for statistical significance was selected to be p<.05 for the primary analysis, as well as for all subgroups analyses and meta-regression. Forest plots were generated separately on Microsoft Excel using previously published methods to aid in presentation of the results [20]. All data including information on the inclusion/exclusion of studies and extraction of data for meta-analysis is available from the corresponding author by request.

## Results

### Included Studies

We included nineteen studies involving a total of 1683 subjects in this meta-analysis [21]–[39]. From the search on Pubmed and related bibliography above described 268 studies were selected. A total of 249 manuscripts were excluded for the following reasons: 123 were not randomized placebo controlled trials, 61 did not examine sleep disorders, 40 did not examine primary sleep disorders, thirteen did not examine the effects of melatonin, four did not have sample size fitting the inclusion criteria, five manuscripts were follow-up studies, two manuscripts were not in a peer-reviewed journal, and one study was retracted. Nineteen studies were included in the analysis, fourteen studies on the efficacy of melatonin for the treatment of insomnia, four studies on delayed sleep phase syndrome and one study on REM sleep behavior disorder. Table 1 depicts the characteristics of included studies in this meta-analysis.

**Table 1:** Characteristics of Included Trials.

Author	Year	Sample Size	Age	Duration	Dose	Design
Wade AG [22]	2011	746	Adults	21 days/182 days	2mg	Parallel
Kunz D [23]	2010	8	Adults	28 days	3 mg	Cross-over
van Geijlswijk IM [24]	2010	70	Children	7 days	0.05 mg/kg, 0.1 mg/kg, 0.15 mg/kg	Parallel
Luthringer R [25]	2009	40	Adults	56 days	2 mg	Parallel
Garzón C [26]	2009	22	Adults	126 days	5 mg	Cross-over
Lemoine P [27]	2007	170	Adults	21 days	2 mg	Parallel
Wade AG [28]	2007	354	Adults	21 days	2 mg	Parallel
Mundey K [29]	2005	13	Adults	28 days	0.3 mg or 3 mg	Parallel
Smits MG [30]	2003	62	Children	28 days	5 mg	Parallel
Almeida Montes LG [31]	2002	10	Adults	21 days	0.3 mg, 1 mg	Cross-over
Kayumov L [32]	2001	22	Adults	28 days	5 mg	Cross-over
Smits MG [33]	2001	40	Children	28 days	5 mg	Parallel
Zhdanova IV [34]	2001	30	Adults	28 days	0.1 mg, 0.3 mg, 1 mg	Cross-over
Dawson D [35]	1998	12	Adults	8 days	0.5 mg	Cross-over
Nagtegaal JE [36]	1998	25	Adults	28 days	5 mg	Cross-over
Ellis CM [37]	1996	15	Adults	7 days	5 mg	Cross-over
Haimov I [38]	1995	26	Adults	7 days	2 mg	Parallel
Dahlitz M [39]	1991	8	Adults	28 days	5 mg	Cross-over
James SP [40]	1989	10	Adults	14 days	1 mg, 5 mg	Cross-over

### Sleep Onset Latency

**Figure 1:**
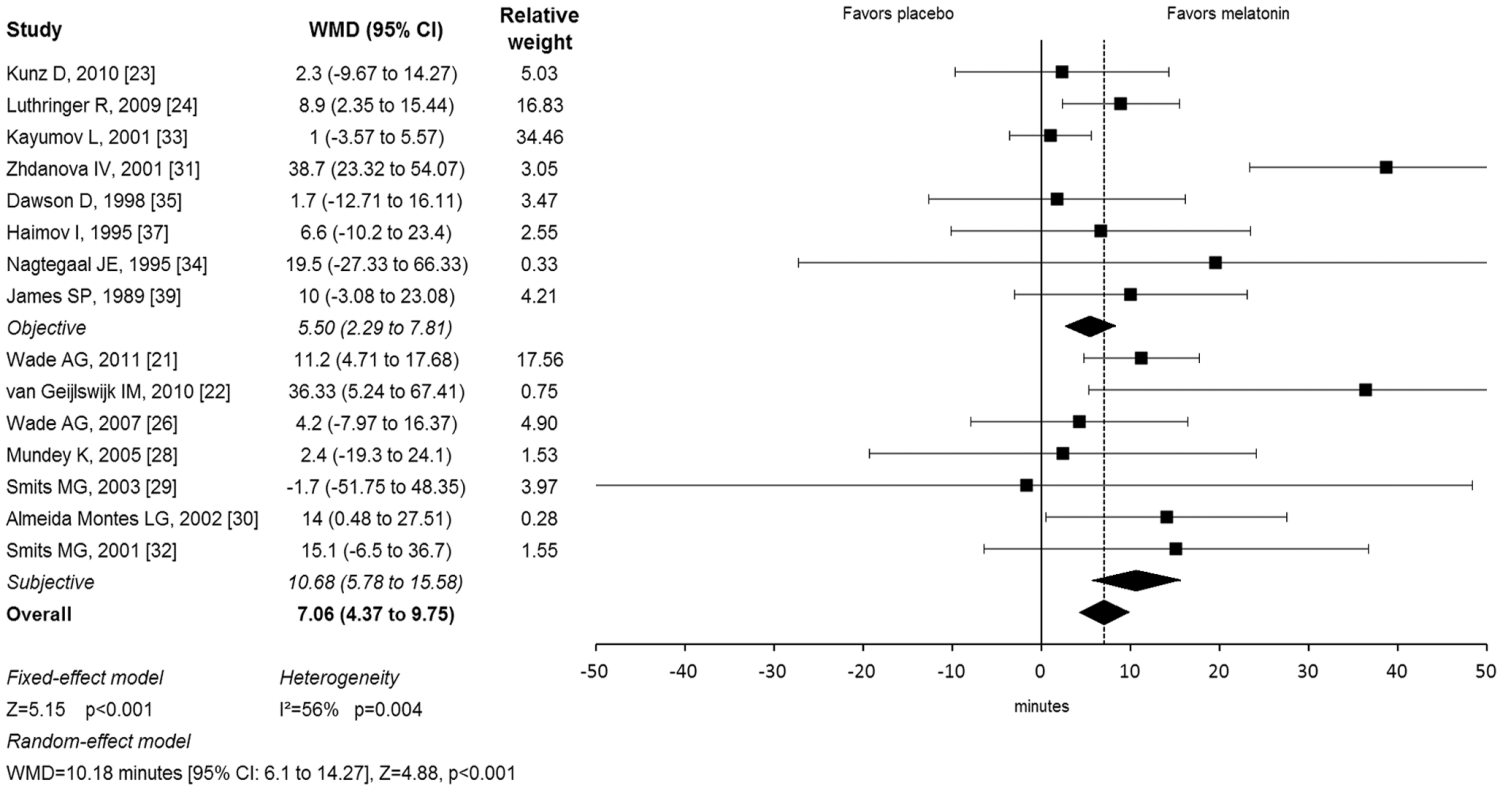
Efficacy of Melatonin in Reducing Sleep Latency. Forest plot depicting reduction of sleep latency in melatonin compared to placebo. Meta-analysis demonstrated a significant benefit of melatonin in reducing sleep latency. WMD = weighted mean difference; CI = confidence interval.

Our meta-analysis demonstrated melatonin had a significant benefit in reducing sleep latency. Subjects randomly assigned to melatonin fell asleep 7 minutes earlier on average than subjects receiving placebo (weighted mean difference (WMD) = 7.06 minutes [95% CI: 4.37 to 9.75], Z = 5.15, p<0.001). Figure 1 illustrates a forest plot depicting the estimated efficacy of melatonin from individual trials. There was significant evidence of heterogeneity between trials (Q = 31.9, df = 14, p = 0.004, I^2^ = 56%). In the random effects model, sleep latency was reduced by over 10 minutes (WMD = 10.18 minutes [95% CI: 6.1 to 14.27], Z = 4.88, p<0.001). We found no significant evidence of publication bias based on the Egger’s Test (intercept = 1.08, [95% CI: −0.35 to 2.52], t = 1.62, p = 0.12). Stratifying trials by objective and subjective measures of sleep onset did not significantly reduce heterogeneity between trials (Q = 3, df = 1, p = 0.08). Melatonin significantly reduced sleep latency on both objective (WMD = 5.50 minutes [95% CI = 2.29 to 8.71], Z = 3.36, p<0.001) and subjective measures (WMD = 10.68 minutes [95% CI: 5.78 to 15.58], z = 4.27, p<0.001). Meta-regression demonstrated that trials of longer duration (parameter estimate (PE) = 0.53 [95% CI = 0.21 to 0.86], p = 0.001) reported greater effects on sleep latency. Trials using higher doses of melatonin also reported greater effects of melatonin on sleep latency at trend levels (PE = 1.95 [95% CI = −0.00 to 3.91], p = 0.05).

### Total Sleep Time

**Figure 2:**
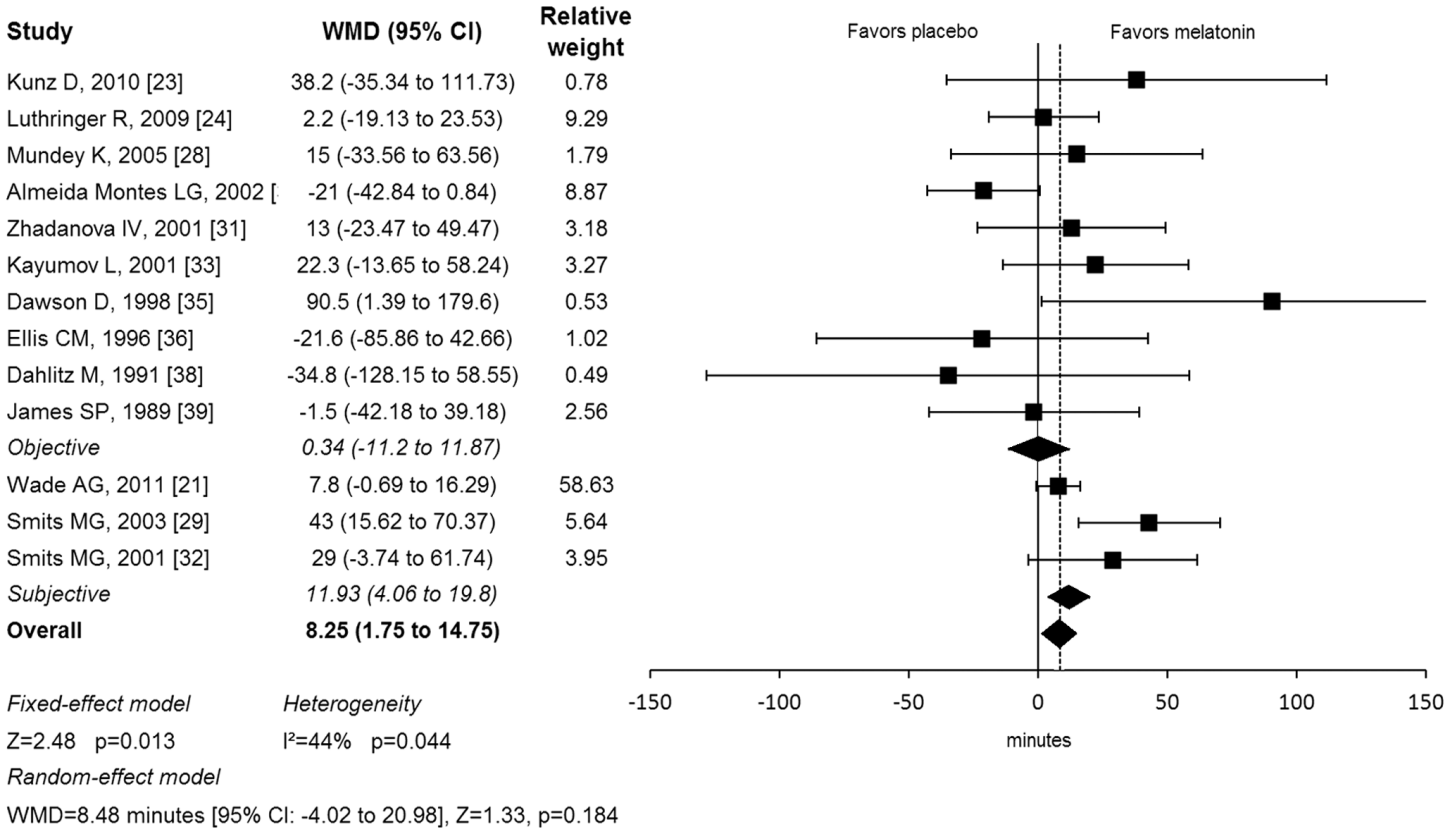
Efficacy of Melatonin in Increasing Total sleep Time. Forest plot depicting change in total sleep time with melatonin compared to placebo treatment. Meta-analysis demonstrated a significant benefit of melatonin in increasing total sleep time. WMD = weighted mean difference; CI = confidence interval.

Melatonin also significantly increased total sleep time compared to placebo. Subjects randomly assigned to melatonin had on average a total sleep time 8 minutes longer than subjects taking placebo (WMD = 8.25 minutes [95% CI: 1.74 to 14.75], Z = 2.48, p = 0.013). Figure 2 illustrates a forest plot depicting the estimated efficacy of melatonin in individual trials. There was significant evidence of heterogeneity between trials (Q = 21.44, df = 12, p = 0.044, I^2^ = 44%). Total sleep time was increased by 8 minutes in the random effects model (WMD = 8.48 minutes [95% CI: −4.02 to 20.98], Z = 1.33, p = 0.184). We found no evidence of publication bias based on the Egger’s Test (intercept = 0.3 [95% CI: −0.9 to 1.7], t = 0.6, p = 0.52). Stratifying trials by whether objective or subjective measures of total sleep time were utilized reduced heterogeneity at trend levels (Q = 2.6, df = 1, p = 0.10). Melatonin significantly increased total sleep time on subjective measures (WMD = 11.93 minutes [95% CI: 4.06 to 19.81], Z = 2.91, p = 0.002) but did not on objective measures (WMD = 0.33 minutes [95% CI: −11.19 to 11.87], Z = 0.05, p = 0.95). Meta-regression demonstrated that trials of longer duration (PE = 1.60 [95% CI: 0.50 to 2.69], p = 0.004) reported greater effects on total sleep time, as well as trials using higher doses of melatonin (PE = 7.25 [95% CI: 1.94 to 12.56], p = 0.007).

### Sleep Quality

**Figure 3:**
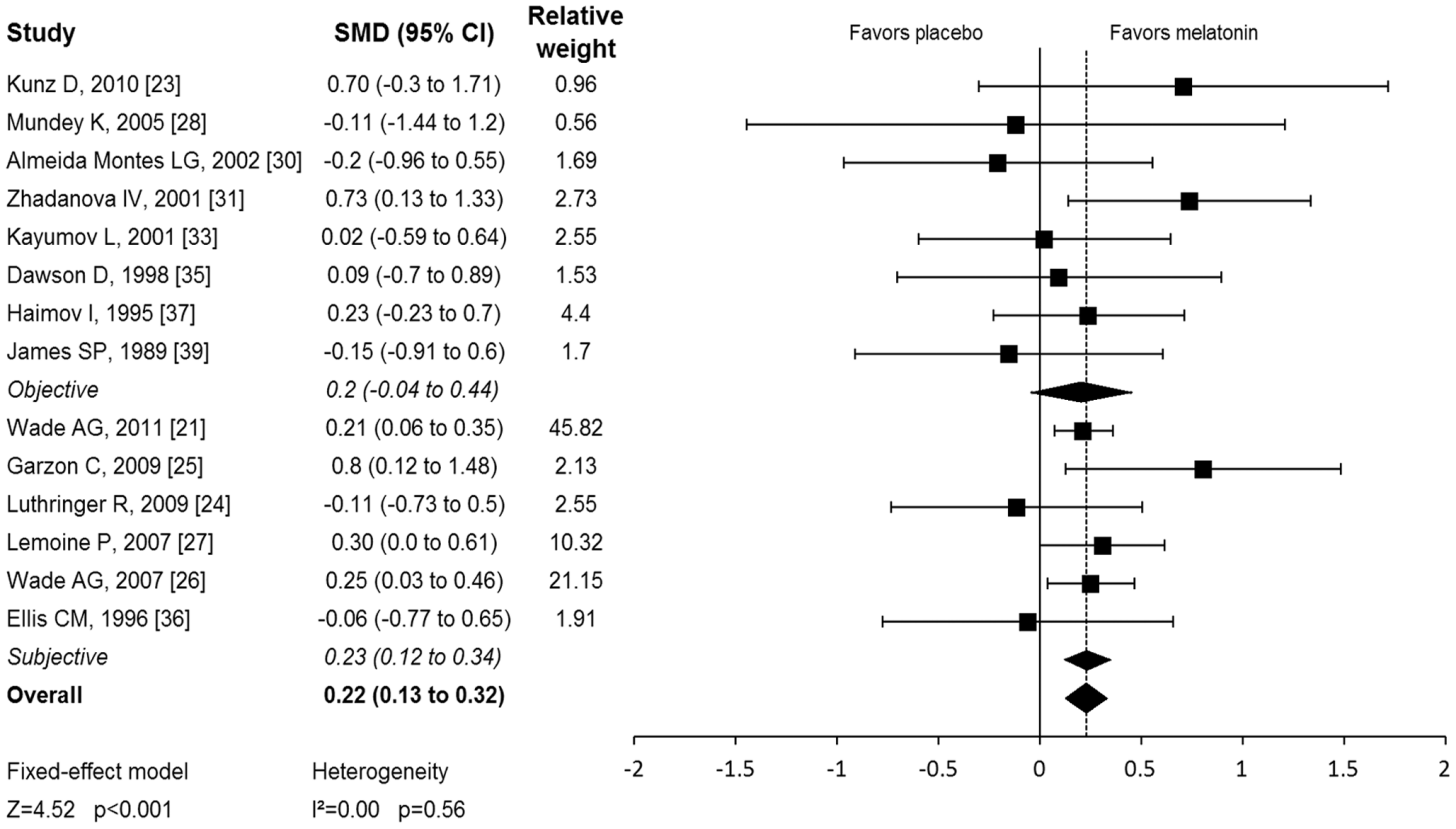
Effect of Melatonin on Sleep quality. Forest plot depicts sleep quality with melatonin compared to placebo. Meta-analysis demonstrated a significant benefit of melatonin in improving sleep quality. SMD = standardized mean difference; CI = confidence interval.

Melatonin demonstrated a significant effect in improving sleep quality. Subjects randomly assigned to melatonin had improvements in sleep quality compared to placebo (standardized mean difference (SMD) = 0.22 [95% CI: 0.12 to 0.32], Z = 4.52, p<0.001). Figure 3 illustrates a forest plot depicting the estimated efficacy of melatonin from individual trials. No significant evidence of heterogeneity between trials was observed (Q = 11.59, df = 13, p = 0.56, I^2^ = 0). A random effects model provided the same overall effect. We found no significant evidence of publication bias based on the Egger’s Test (intercept = −0.13 [95% CI: −1.08 to 0.81], t = 0.30, p = 0.76). Stratifying trials by objective and subjective measures of sleep quality did not reduce heterogeneity between trials (Q = 0.05, df = 1, p = 0.82). Melatonin improved sleep quality to a similar degree on both subjective (SMD = 0.23 [95% CI: 0.12 to 0.34], Z = 4.23, p<0.001) and objective measures (SMD = 0.20 [95% CI: −0.04 to 0.44], Z = 1.61, p = 0.10). Meta-regression demonstrated no significant effects of trial duration (PE = 0.005 [95% CI: −0.0006 to 0.012], p = 0.08) or dose of melatonin on sleep quality (PE = 0.011 [95% CI: −0.114 to 0.090], p = 0.81).

## Discussion and Conclusion

Our meta-analysis demonstrates that melatonin significantly improves sleep in subjects with primary sleep disorders compared to placebo. Melatonin reduces sleep-onset latency, increases total sleep time and improves overall sleep quality compared to placebo to a statistically significant degree. It should be noted that the improvements in sleep parameters in absolute terms were smaller than previous meta-analyses of benzodiazepines and newer non-benzodiazepine sleep medications. For instance, the reduction in sleep latency observed with melatonin in this meta-analysis, slightly less than 7 minutes, was less than sleep-latency reduction observed in previous meta-analyses of other available sleep medications. A previous meta-analysis demonstrated a significant benefit of benzodiazepines (10.0 to 19.6 minutes) and non-benzodiazepine sleep medicines (12.8 to 17 minutes) in reducing sleep-onset latency for primary sleep disorders [21]. Thus prescription sleep medications are quite likely more effective than melatonin, although head-to-head trials could definitely alleviate any doubts regarding the relative efficacy of these agents.

Meta-regressions were performed to assess the relationship between effect, duration and dose. Higher melatonin doses and longer duration trials were related to significant greater effect sizes on sleep latency and total sleep time. These findings suggest that there is no evidence of the development of tolerance with melatonin use. This stands in contrast to other commonly used hypnotics such as benzodiazepines [13]. No greater effects in sleep quality were observed with melatonin dose or trial duration, suggesting that melatonin effects on sleep quality are constant for any duration or dose.

Given our findings, it is important to note some limitations of this meta-analysis. Due to the relatively small number of trials that met our inclusion criteria, our meta-regression analysis had limited power. This problem was exacerbated by one trial that contributed a very large weight to our overall findings [22]. The relatively small number of trials also limits the ability of the Egger’s Test to demonstrate publication bias. However there was no evidence of publication bias for all outcome measures on funnel plot as well. There were relatively few studies examining the efficacy of melatonin in children with primary sleep disorders and this precluded any stratified subgroup analysis based on age. The presence of mostly trials examining primary insomnia limited our ability to perform an analysis based on diagnoses.

Despite these limitations, this meta-analysis demonstrated that exogenous melatonin administered to subjects with primary sleep disorders modestly improved sleep parameters including sleep latency, total sleep time and sleep quality. This finding corroborates the results of a previous meta-analysis conducted in the area several years ago that also demonstrated a significant benefit of melatonin [17]. The benefits of melatonin compared to placebo appear smaller than that of available prescription sleep medications. However, melatonin should be considered in clinical practice due to its benign side-effect profile, cost and limited evidence of habituation and tolerance. Further research is needed to examine the long-term benefits of sleep medications including the comparative efficacy of melatonin to common prescription sleep medication.

## Supporting Information

Document S1 **PRISMA 2009 Checklist.** Checklist showing what page are each characteristic analyzed.
(DOC)

Document S2 **PRISMA 2009 Flow Chart.** Flow Diagram. Flow chart showing the selection of studies for this review.
(DOC)

## References

[1] American Psychiatric Association (2000) Diagnostic and Statistical Manual of Mental Disorders - Text Revised (DSM-IV-TR).

[2] National Sleep Foundation (2005) Sleep in America Poll. Washington DC: National Sleep Foundation. 24 p.

[3] Hossain JL, Shapiro CM (2002) The prevalence, cost implications, and management of sleep disorders: an overview. Sleep Breath 6: 85–102. 12075483 10.1007/s11325-002-0085-1

[4] Dikeos D, Georgantopoulos G (2011) Medical comorbidity of sleep disorders. Curr Opin Psychiatry 24: 346–354. 21587079 10.1097/YCO.0b013e3283473375

[5] Ozminkowski RJ, Wang S, Walsh JK (2007) The direct and indirect costs of untreated insomnia in adults in the United States. Sleep 30: 263–273. 17425222 10.1093/sleep/30.3.263

[6] Ellen RL, Marshall SC, Palayew M, Molnar FJ, Wilson KG, et al (2006) Systematic review of motor vehicle crash risk in persons with sleep apnea. J Clin Sleep Med 2: 193–200. 17557495

[7] Gallicchio L, Kalesan B (2009) Sleep duration and mortality: a systematic review and meta-analysis. J Sleep Res 18: 148–158. 19645960 10.1111/j.1365-2869.2008.00732.x

[8] Leger D, Guilleminault C, Bader G, Levy E, Paillard M (2002) Medical and socio-professional impact of insomnia. Sleep 25: 625–629. 12224841

[9] Morgan K, Kucharczyk E, Gregory P (2011) Insomnia: evidence-based approaches to assessment and management. Clin Med 11: 278–281. 10.7861/clinmedicine.11-3-278 PMC4953326 21902086

[10] Morin CM, Culbert JP, Schwartz SM (1994) Nonpharmacological interventions for insomnia: a meta-analysis of treatment efficacy. Am J Psychiatry 151: 1172–1180. 8037252 10.1176/ajp.151.8.1172

[11] Murtagh DR, Greenwood KM (1995) Identifying effective psychological treatments for insomnia: a meta-analysis. J Consult Clin Psychol 63: 79–89. 7896994 10.1037//0022-006x.63.1.79

[12] Smith MT, Perlis ML, Park A, Smith MS, Pennington J, et al (2002) Comparative meta-analysis of pharmacotherapy and behavior therapy for persistent insomnia. Am J Psychiatry 159: 5–11. 11772681 10.1176/appi.ajp.159.1.5

[13] Srinivasan V, Brzezinski A, Pandi-Perumal SR, Spence DW, Cardinali DP, et al (2011) Melatonin agonists in primary insomnia and depression-associated insomnia: are they superior to sedative-hypnotics? Prog Neuropsychopharmacol Biol Psychiatry 35: 913–923. 21453740 10.1016/j.pnpbp.2011.03.013

[14] Morin CM (2005) Psychological and behavioral treatments for primary insomnia. In: Kryger MH, Roth T, Dement WC, editors. Principles and practice of sleep medicine. Fourth ed. Philadelphia: Elsevier Inc. 726–737.

[15] Claustrat B, Brun J, Chazot G (2005) The basic physiology and pathophysiology of melatonin. Sleep Med Rev 9: 11–24. 15649735 10.1016/j.smrv.2004.08.001

[16] Bliwise DL, Ansari FP (2007) Insomnia associated with valerian and melatonin usage in the 2002 National Health Interview Survey. Sleep 30: 881–884. 17682659 10.1093/sleep/30.7.881 PMC1978376

[17] Buscemi N, Vandermeer B, Hooton N, Pandya R, Tjosvold L, et al (2005) The efficacy and safety of exogenous melatonin for primary sleep disorders. A meta-analysis. J Gen Intern Med 20: 1151–1158. 16423108 10.1111/j.1525-1497.2005.0243.x PMC1490287

[18] Egger M, Davey Smith G, Schneider M, Minder C (1997) Bias in meta-analysis detected by a simple, graphical test. BMJ 315: 629–634. 9310563 10.1136/bmj.315.7109.629 PMC2127453

[19] Deeks J, Higgins J, Altman D (2003) Cochrane Reviewers’ Handbook 4.2.1. John Wiley & Sons, Ltd.

[20] Neyeloff JL, Fuchs SC, Moreira LB (2012) Meta-analyses and Forest plots using a microsoft excel spreadsheet: step-by-step guide focusing on descriptive data analysis. BMC Res Notes 5: 52. 22264277 10.1186/1756-0500-5-52 PMC3296675

[21] Buscemi N, Vandermeer B, Friesen C, Bialy L, Tubman M, et al (2007) The efficacy and safety of drug treatments for chronic insomnia in adults: a meta-analysis of RCTs. J Gen Intern Med 22: 1335–1350. 17619935 10.1007/s11606-007-0251-z PMC2219774

[22] Wade AG, Crawford G, Ford I, McConnachie A, Nir T, et al (2011) Prolonged release melatonin in the treatment of primary insomnia: evaluation of the age cut-off for short- and long-term response. Curr Med Res Opin 27: 87–98. 21091391 10.1185/03007995.2010.537317

[23] Kunz D, Mahlberg R (2010) A two-part, double-blind, placebo-controlled trial of exogenous melatonin in REM sleep behaviour disorder. J Sleep Res 19: 591–596. 20561180 10.1111/j.1365-2869.2010.00848.x

[24] van Geijlswijk IM, van der Heijden KB, Egberts AC, Korzilius HP, Smits MG (2010) Dose finding of melatonin for chronic idiopathic childhood sleep onset insomnia: an RCT. Psychopharmacology (Berl) 212: 379–391. 20668840 10.1007/s00213-010-1962-0 PMC2952772

[25] Luthringer R, Muzet M, Zisapel N, Staner L (2009) The effect of prolonged-release melatonin on sleep measures and psychomotor performance in elderly patients with insomnia. Int Clin Psychopharmacol 24: 239–249. 19584739 10.1097/YIC.0b013e32832e9b08

[26] Garzon C, Guerrero JM, Aramburu O, Guzman T (2009) Effect of melatonin administration on sleep, behavioral disorders and hypnotic drug discontinuation in the elderly: a randomized, double-blind, placebo-controlled study. Aging Clin Exp Res 21: 38–42. 19225268 10.1007/BF03324897

[27] Lemoine P, Nir T, Laudon M, Zisapel N (2007) Prolonged-release melatonin improves sleep quality and morning alertness in insomnia patients aged 55 years and older and has no withdrawal effects. J Sleep Res 16: 372–380. 18036082 10.1111/j.1365-2869.2007.00613.x

[28] Wade AG, Ford I, Crawford G, McMahon AD, Nir T, et al (2007) Efficacy of prolonged release melatonin in insomnia patients aged 55–80 years: quality of sleep and next-day alertness outcomes. Curr Med Res Opin 23: 2597–2605. 17875243 10.1185/030079907X233098

[29] Mundey K, Benloucif S, Harsanyi K, Dubocovich ML, Zee PC (2005) Phase-dependent treatment of delayed sleep phase syndrome with melatonin. Sleep 28: 1271–1278. 16295212 10.1093/sleep/28.10.1271

[30] Smits MG, van Stel HF, van der Heijden K, Meijer AM, Coenen AM, et al (2003) Melatonin improves health status and sleep in children with idiopathic chronic sleep-onset insomnia: a randomized placebo-controlled trial. J Am Acad Child Adolesc Psychiatry 42: 1286–1293. 14566165 10.1097/01.chi.0000085756.71002.86

[31] Almeida Montes LG, Ontiveros Uribe MP, Cortes Sotres J, Heinze Martin G (2003) Treatment of primary insomnia with melatonin: a double-blind, placebo-controlled, crossover study. J Psychiatry Neurosci 28: 191–196. 12790159 PMC161743

[32] Kayumov L, Brown G, Jindal R, Buttoo K, Shapiro CM (2001) A randomized, double-blind, placebo-controlled crossover study of the effect of exogenous melatonin on delayed sleep phase syndrome. Psychosom Med 63: 40–48. 11211063 10.1097/00006842-200101000-00005

[33] Smits MG, Nagtegaal EE, van der Heijden J, Coenen AM, Kerkhof GA (2001) Melatonin for chronic sleep onset insomnia in children: a randomized placebo-controlled trial. J Child Neurol 16: 86–92. 11292231 10.1177/088307380101600204

[34] Zhdanova IV, Wurtman RJ, Regan MM, Taylor JA, Shi JP, et al (2001) Melatonin treatment for age-related insomnia. J Clin Endocrinol Metab 86: 4727–4730. 11600532 10.1210/jcem.86.10.7901

[35] Dawson D, Rogers NL, van den Heuvel CJ, Kennaway DJ, Lushington K (1998) Effect of sustained nocturnal transbuccal melatonin administration on sleep and temperature in elderly insomniacs. J Biol Rhythms 13: 532–538. 9850013 10.1177/074873098129000354

[36] Nagtegaal JE, Kerkhof GA, Smits MG, Swart AC, Van Der Meer YG (1998) Delayed sleep phase syndrome: A placebo-controlled cross-over study on the effects of melatonin administered five hours before the individual dim light melatonin onset. J Sleep Res 7: 135–143. 9682186 10.1046/j.1365-2869.1998.00102.x

[37] Ellis CM, Lemmens G, Parkes JD (1996) Melatonin and insomnia. J Sleep Res 5: 61–65. 8795804 10.1046/j.1365-2869.1996.00003.x

[38] Haimov I, Lavie P, Laudon M, Herer P, Vigder C, et al (1995) Melatonin replacement therapy of elderly insomniacs. Sleep 18: 598–603. 8552931 10.1093/sleep/18.7.598

[39] Dahlitz M, Alvarez B, Vignau J, English J, Arendt J, et al (1991) Delayed sleep phase syndrome response to melatonin. Lancet 337: 1121–1124. 1674014 10.1016/0140-6736(91)92787-3

[40] James SP, Sack DA, Rosenthal NE, Mendelson WB (1990) Melatonin administration in insomnia. Neuropsychopharmacology 3: 19–23. 2306332

