# Supplementary material for: Meta-Analysis: Melatonin for the Treatment of Primary Sleep Disorders
Source: PLoS One. 2013 May 17;8(5):e63773. doi: 10.1371/journal.pone.0063773 (PMC3656905; doi:10.1371/journal.pone.0063773)
Supplement: Document S2 — PRISMA 2009 Flow Chart. Flow Diagram. Flow chart showing the selection of studies for this review. (DOC) [file pone.0063773.s002.doc]

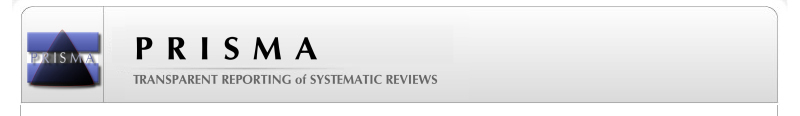
**PRISMA 2009 Flow Diagram**

**Screening**

**Included**

**Eligibility**

**Identification**

Records identified through database searching
(n =118)

Additional records identified through other sources
(n =150)

Records after duplicates removed
(n =268 )

Records screened
(n =268)

Records excluded
(n =0)

Full-text articles assessed for eligibility
(n =268)

Full-text articles excluded, with reasons
(n =249)

Studies included in qualitative synthesis
(n =19)

Studies included in quantitative synthesis (meta-analysis)
(n =19)
